# Evolution of Brains and Computers: The Roads Not Taken

**DOI:** 10.3390/e24050665

**Published:** 2022-05-09

**Authors:** Ricard Solé, Luís F. Seoane

**Affiliations:** 1 ICREA-Complex Systems Lab, Universitat Pompeu Fabra, Dr Aiguader 88, 08003 Barcelona, Spain; 2 Institut de Biologia Evolutiva, CSIC-UPF, Pg Maritim de la Barceloneta 37, 08003 Barcelona, Spain; 3 Santa Fe Institute, 1399 Hyde Park Road, Santa Fe, NM 87501, USA; 4 Departamento de Biología de Sistemas, Centro Nacional de Biotecnología (CSIC), C/Darwin 3, 28049 Madrid, Spain; lf.seoane@cnb.csic.es; 5 Grupo Interdisciplinar de Sistemas Complejos (GISC), 28049 Madrid, Spain

**Keywords:** evolution, brains, deep learning, embodiment, neural networks, artificial intelligence, neurorobotics

## Abstract

When computers started to become a dominant part of technology around the 1950s, fundamental questions about reliable designs and robustness were of great relevance. Their development gave rise to the exploration of new questions, such as what made brains reliable (since neurons can die) and how computers could get inspiration from neural systems. In parallel, the first artificial neural networks came to life. Since then, the comparative view between brains and computers has been developed in new, sometimes unexpected directions. With the rise of deep learning and the development of connectomics, an evolutionary look at how both hardware and neural complexity have evolved or designed is required. In this paper, we argue that important similarities have resulted both from convergent evolution (the inevitable outcome of architectural constraints) and inspiration of hardware and software principles guided by toy pictures of neurobiology. Moreover, dissimilarities and gaps originate from the lack of major innovations that have paved the way to biological computing (including brains) that are completely absent within the artificial domain. As it occurs within synthetic biocomputation, we can also ask whether alternative minds can emerge from A.I. designs. Here, we take an evolutionary view of the problem and discuss the remarkable convergences between living and artificial designs and what are the pre-conditions to achieve artificial intelligence.

## 1. Introduction

With the evolution of life came cognition [1]. As soon as cells were able to evolve into autonomous agents, the combination of receptors gathering signals and mechanisms of response to those signals rapidly transformed into rich molecular networks. Those networks provided the basis for the smaller scale of computation: survival requires exploiting resources in a reliable way that allows reproduction. Since this is a combination of growing and being robust against fluctuations over a minimal time window, computation was tied to predictive power [2,3,4,5]. It is this power what actually might foster the evolution towards brains [6], large and small: in order to reduce the uncertainty of external fluctuations, prediction is a convenient faculty. If we follow the steps towards cognitive complexity that predate the emergence of brains, several key ingredients seem necessary. Looking at their evolutionary emergence is relevant for our discussion concerning the space of possible cognitive networks. One of them was the invention of neurons: specialized cell types with a marked elongated, branched shape capable of establishing connections. In most cases, these are polar, unidirectional structures, with response functions that involve nonlinear thresholds. The power of neurons became a reality as soon as groups of them became interconnected, leading to the first neural networks. Among the key innovations associated to these early assemblies, interneurons must have been a crucial step towards information processing beyond the sensor–actuator chain. With the Cambrian explosion of life, the rise of animals favored the development of sensory organs, learning, and movement [7].

All these factors came together within a novel developmental design: brains emerged within bilateral animals, and those newcomers actively explored their worlds, moving around. A compelling proposal concerning the origins of brains is, in fact, the so-called *moving hypothesis*: it posits that the active exploration of the external world fostered the evolutionary path that led to brains [6]. In a novel biosphere dominated by predator–prey arms races, brains were an optimal solution to deal with information. If we fast-forward in time, several important changes took place paving the way towards complex brains. This is particularly dramatic for human brains: a rapid expansion during evolution facilitated the addition of microcircuit modules to a multilayered neocortex [8].

**Figure 1:**
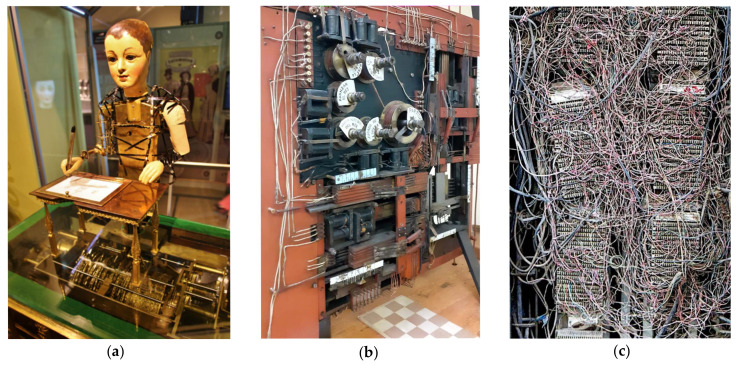
Technological metaphors used to describe diverse aspects of cognitive complexity before electronic computers. (**a**) Mechanical automata able to write using a set of connected gears that could be changed to execute diverse writing or drawing tasks. (**b**) Leonardo Torres-Quevedo 1910-prototype of his electromechanical chess-playing automaton. (**c**) Tangled network of interconnections in a telephone network board.

Turning our attention to machines, we can see how inventors and scholars have repeatedly drawn inspiration from nature’s cognitive systems. Sometimes through outright imitation, as in the case of mechanical automata (Figure 1a). Others by focusing efforts on human-specific cognitive problems (e.g., chess, Figure 1b) [9]. In yet other cases, through converging metaphors—e.g., from Cajal’s flows within neurons and across neural circuits to technological networks that enable the flow of information (Figure 1c). The exchange of ideas between computational designs and theoretical neuroscience has been constant. Prediction too has been a major force in the development of a large part of technology, particularly after the rise of Information Technology from the 1950s [10]. In parallel with the development of the theory of computation, the first steps towards a theory of neural networks came to life, starting from early comparisons between brains and computers.

The first computers were plagued with problems associated to faulty units: vacuum tubes were prone to failure. Far from the reliable nature of brains, where neurons can die with no major consequences for the system-level performance, single-element failures could cause large disruptions. The comparative analysis between brains and computers (or computational analogies of brains) has been a recurrent topic since von Neumann’s book *The computer and the brain* [11]. In the original formulation, the main problem was how to design reliable computers made of unreliable parts. Such approximation was largely forgotten within computer science with the rise of integrated circuits, although the problem became a central topic in the domain of neural networks. With the potential of *simulating* neural systems some of the early metaphors of memory involved strong analogies with magnetic materials. Such analogies would be developed in depth with the use of the statistical physics of spin glasses as computational substrates [12,13]. These similarities eventually provided the basis for the attractor picture that is now widespread.

Over the last decade, a new wave of excitement has emerged with the rise of Deep Learning networks [14]. These descendants of the early multilayer neural networks developed in the 1990s have been accompanied with a considerable set of expectations (and hype). Because of their remarkable power to deal with specific problems far beyond the capacities of humans, claims have been repeatedly made suggesting that larger systems will eventually achieve cognitive skills similar (if not greater) than human brains, including consciousness or awareness. (See the recent stir caused by OpenAI’s chief scientist, Ilya Sutskever, claiming that “it may be that today’s large neural networks are slightly conscious”. https://lastweekin.ai/p/conscious-ai?s=r, accessed on 2 May 2022). However, as it has happened before many times (the winter–spring cycles of A.I.), artificially intelligent systems are still rather far from our general intelligence [15], and, indeed, they often appear brittle when taken even slightly out of their well-controlled closed worlds [16].

All this mimicry, inspiration, and convergences bear some pressing questions: do natural cognitive designs exhaust the space of the possible? Will every artificial cognitive solution ever found correspond to an earlier invention in nature? If so, then understanding natural cognition should be sufficient to learn everything that there is to know about intelligence and cognition. Might comprehending nature be necessary as well—i.e., does every cognitive solution respond to some natural challenge or feature that we need to understand in order to ultimately grasp cognition? If so, which is a minimal set of such challenges and features that can generate the range of cognitive designs? It is also possible that nature is not so restrictive and all-encompassing. This would leave a large elbow room for artificial cognition, human invention, and open-ended progress. Yet, more wondrous questions are also put forward: what solutions might have been missed in the natural history of cognition? Are there artificial cognitive designs that cannot be reached by extant evolutionary forces alone?

In this paper, we argue that very relevant lessons can (and must) be obtained from a comparative analysis between evolved and designed cognitive systems. On the one hand, there are several non-trivial observations that suggest a limited repertoire of design principles that pervade and constrain the space of the possible: evolved and artificial architectures often converge. Secondly, there is a question regarding certain dynamical patterns exhibited by living systems that seldom appear in artificial neural networks. Brains seem to operate close to critical states: is this a relevant trait to be considered when building artificial counterparts? Third, we will consider a list of attributes of human brains that define a gap between our species and any other living organism and we will see why A.I. systems might require to include evolutionary dynamics to get there.

## 2. Contingent versus Convergent Evolution

Digital culture historian Kevin Kelly suggested in an essay on A.I. that future developments would allow us to create “artificial aliens” [17]. Specifically, Kelly conjectured that ongoing developments within this field will eventually create the conditions for new kinds of intelligences different from human ones:

*Some traits of human thinking will be common (as common as bilateral symmetry, segmentation, and tubular guts are in biology), but the possibility space of viable minds will likely contain traits far outside what we have evolved. It is not necessary that this type of thinking be faster than humans, greater, or deeper. In some cases it will be simpler. Our most important machines are not machines that do what humans do better, but machines that can do things we can’t do at all. Our most important thinking machines will not be machines that can think what we think faster, better, but those that think what we can’t think.*

Such possibility is, from an evolutionary perspective, very appealing. The problem of how cognitive complexity emerged is a difficult one because behavior does not leave fossils (except in some limited and indirect fashion) and little can be said about intelligence. In this context, an alternative approach to standard comparative and phylogenetic approaches would be the study of “synthetic minds” resulting from engineering neural networks or evolvable robots [18]. In a nutshell, by designing or evolving artificial alternatives to living matter, it could be possible perhaps to recreate the conditions for minds (and even consciousness) to emerge. In principle, it can be argued that multiple possibilities, perhaps including these “alien” minds pointed out by Kelly, might be found. Is that the case? Is there a space of endless possibilities inhabited by artificial intelligences orthogonal to those found in nature?

Two extreme possibilities can be envisaged. In one, consistent with Kelly’s picture, completely new forms of intelligence might be possible. This situation fits the picture of evolutionary change as a highly contingent process with many potential paths available. Contingency was particularly advocated by the late Stephen J. Gould [19] who suggested that, if we would be able to re-run the tape of evolution, a completely different biosphere (and different minds) would be obtained. The human brain actually tells a story of tinkering associated to its nested origins.

However, the study of development reveals that very strong constraints might deeply limit the potential paths that can be followed. This is illustrated for example by the architecture of camera eyes that are found across many biological groups, from some single-cell organisms or jellyfish to cephalopods or vertebrates. A remarkable design principle is always at work despite their totally independent origins [20]. If we look at the evolution of cognition in nature, what do we see? Do minds converge?

A remarkable observation from a comparative analysis of brain structures is that radically different topologies seem to share very similar functionalities [21]. A perfect illustration is provided by birds versus mammalian brains. Their early neural organization diverged 340 Myr ago, evolving in completely independent ways. And yet, their obviously different neural structures do not generate radically different minds [21,22,23]. Such a convergence of minds is supported by the common traits of behavioral patterns, such as associative learning, predator avoidance or decision making mechanisms that indicate the presence of a common cognitive toolkit [24,25]. These commonalities in terms of cognitive repertoires could in fact be shared by aneural systems [26]. Divergent architectural brain designs are found all over the tree of life. Dolphins, for example, have brains that depart from primate ones, showing a combination of archaic features combined with a massively expanded cortex [27]. However, despite the differences, they display complex intelligence, communication skills, and social life. Similarly, the brains of octopuses (members of the class of cephalopods that includes squids and cuttlefish) provide a further instance of convergent traits despite being invertebrates [28]. This group has evolved brains with multilayered cortical maps as well as a unique 8-fold extra neural cluster that autonomously controls the arms. Because of their shape-shifting nature and distributed neural autonomy of the arms, these organisms have been often labeled as “alien” but they perform cognitive tasks similar to those displayed by other animals.

Behind each evolutionary convergence, we can often find shared external pressures (e.g., the need to deal with a same niche) or, more fundamentally for cognition, a deep mathematical principle or constraint. Such key operating principles demand that certain solutions are found over and again by biological brains and their technological counterparts. Let us study some of these commonalities and the crucial computational principles that bring about such design convergences.

### 2.1. Threshold Units

The new wave of computing machines towards the mid 20th century provided the right technological context to simulate logic elements similar to those present in nervous systems [29]. Theoretical developments within mathematical biology by Warren McCulloch and Walter Pitts revealed one first major result: it is possible to define units of cognition (*neurons*) under a logical framework [30,31,32]. These formal neurons were described in terms of threshold units, largely inspired by the state-of-the-art knowledge of real excitable cells. Not surprisingly, the early works of Walter and Pitts had to do with threshold neurons and their mathematical description [33]. Over the last decades, major quantitative advances have been obtained by using a combination of neuron-inspired models with multilayer architecture and novel hardware improvements combined with massive use of training data. The growing understanding of single-neuron dynamics suggests that deeper computational complexity might be at work [34]—but let us focus for a moment on the simplest models.

**Figure 2:**
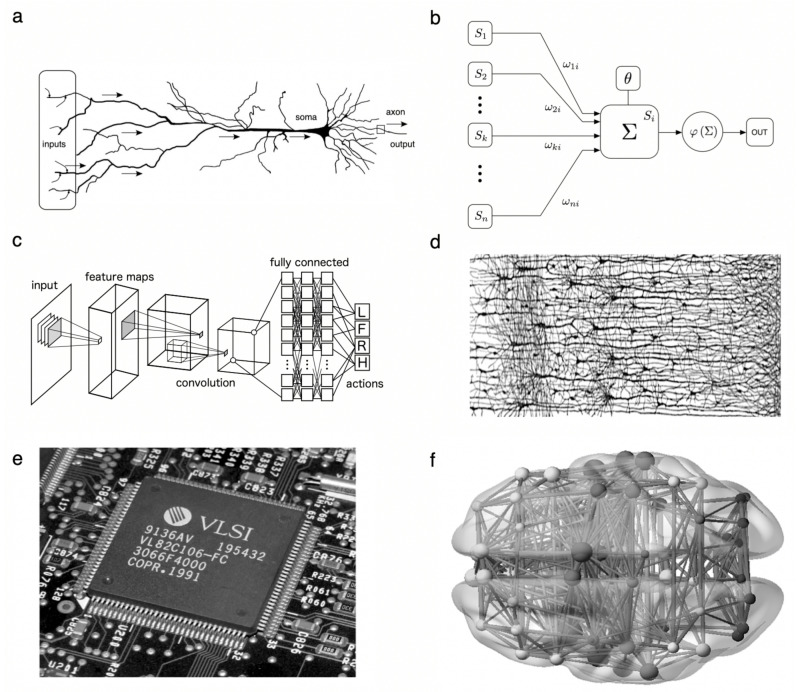
Convergent design principles in living and computational systems. In (**a**), a pyramidal neuron is shown, to be compared with the toy model of a neuron, as suggested by McCulloch and Pitts (**b**) where the minimal components are preserved at the logic level. Here, a set of “input neurons” $S_{1},\ldots ,S_{n}$ send their signals to neuron $S_{i}$ where the sum Σ of all inputs, weighted by their specific links $\omega_{ki}$, is performed and compared with an internal threshold number $\theta_{i}$. The decision of firing or not is then made by means of a threshold function φ. Complex tasks can be achieved by using layered ANN structures, which are characteristic of Deep networks (**c**) resembling those found in the brain cortex (**d**). In both VLSI circuits (**e**) and brain connectomes (**f**), a nested hierarchy has been shown to exist, displaying common statistical laws, such as Rent’s rule. This rule establishes that the amount of connections *C* between elements in a sub-system of size *N* with the rest of the system scales as a power law $C∼N^{p}$, with p∼0.8 in both neural and VLSI circuits.

The pictorial conceptualization behind the McCulloch–Pitts model is sketched in Figure 2a,b. The *formal neuron* shown here (Figure 2b) is a simple Boolean system. Its state, $S_{i}\in \Sigma$, takes one of two values: $S_{i}\in \Sigma \equiv \left\{0,1\right\}$ (a description of neurons as spins, $S_{i}\in \Sigma \equiv \left\{-1,+1\right\}$, is often convenient to derive potential energies for formal neural circuits). These two states are commonly associated to neurons resting (inactive) or firing (sending signals to others). Formal neurons react to incoming signals from a set of *N* presynaptic units. Its response is a sudden activation if a weighted sum of the inputs is larger than a threshold [35,36,37]. While activation is all-or-nothing, weights, $\omega_{ij}$, are continuous and tell us how much the state of a presynaptic neuron *j* affects postsynaptic neuron *i* (thus modeling the strength of connections). They can also be positive or negative, hence implementing excitation and inhibition. In the McCulloch–Pitts approach, postsynaptic neuron $S_{i}$ integrates incoming signals as:

(1) $$\begin{array}{ccc} S_{i}\left(t+1\right) & = & \sigma \left(\sum_{j=1}^{N}\omega_{ij}S_{j}\left(t\right)-\theta_{i}\right). \end{array}$$

The additional parameter, $\theta_{i}$, defines the neuron’s threshold. The nonlinear function σ(x) is 1 if its argument is positive and 0 otherwise. Thus, $S_{i}$ fires if the weighted sum of presynaptic inputs is larger than its threshold. The nonlinearity introduced by σ(·) implements the all-or-none neural response. Alternative implementations use smooth step functions—e.g., σ(x)=1/(1+exp(−βx)), where β (an inverse temperature) controls how much the nonlinearity approaches the step function as β→∞.

McCulloch and Pitts crucially showed that formal threshold neurons can build any logic Boolean circuit. A direct consequence is that brains, or at least their Boolean representation, can execute at least any logic operation that computers can perform. The elegant picture emerging from the McCulloch–Pitts model of a formal neuron is a powerful one. They broke new ground by showing that there was a neural analog to logic circuits and provide an important message concerning the power of brains as computational systems. Is this enough to get close to the complexity of brains? The development of ANN has revealed the enormous potential of so-called semi-symbolic artificial intelligence, but their achievements are only a tiny subset of the possible.

Are there alternative ways of designing cognitive networks that are not grounded in threshold-like units? This is a particularly relevant question, since advances in cognitive neuroscience and in particular artificial neural networks are indeed inspired by basic units exhibiting such kind of behavior. There is in fact another instance of threshold-like networks that have evolved in living systems: the webs of gene–gene interactions that rule the dynamics of cells. These so-called gene regulatory networks have also a long tradition that starts in the aftermath of cybernetics and is grounded in a picture of gene–gene interactions similar to the McCulloch–Pitts model [38,39,40,41]. Here, too, information exchanges are mediated by mechanisms that have a different molecular nature but share a fundamental commonality: responses are typically mediated by threshold-like response functions.

### 2.2. Hierarchical Processing of Sensory Inputs

The second half of the 20th century saw quick advances regarding natural visual processing—from Hubel and Wiesel’s identification of V1 neurons responding to light bars tilted at different angles [42,43], to our modern understanding of visual cortical regions meshed in a complex network of hierarchical and parallel processes [44,45,46]. We now have a rather complete view of how static visual stimuli are reconstructed: in the retina, edges are localized by ganglion cells that integrate information about spatial gradients of light [47,48]. This continues in the primary visual systems, where different neurons (such as the ones discovered by Hubel and Wiesel) respond to bars of light of different sizes and at different locations across the visual field. These are building blocks of visual percepts that are later assembled, in higher visual cortices, into geometrical shapes and, eventually (as color is added), into the refined objects that we perceive.

This process is paralleled by modern technology. By design, edge detection has long been mimicked by filters for image processing [49,50], and a hierarchical organization was capitalized by early ANN [51]—a choice inherited by state-of-the-art Convolutional Neural Networks (CNN) [52,53]. Not so much by design, learning algorithms shape the computational roles and organization of layers across these hierarchies, often leading to the convergence of individual receptor fields, e.g., of biological neurons and units of parallel computer vision systems [54]. Some modern CNN show a broader convergence with the biological visual processing pathway [55,56,57,58]. After CNN were trained in object recognition tasks, individual units were identified whose activity correlated with that of real neurons in the ventral visual stream of humans performing the same task. This suggests that both natural and artificial systems converge on some computational steps necessary for visual recognition. Similar convergences are observed for auditory processing, as activity of CNN trained on auditory tasks can predict neural activity in the corresponding cortices [59], or for networks that predict fMRI and MEG responses during language processing tasks [60]. Cutting-edge developments in artificial visual systems incorporate ideas from natural language processing such as transformers or local context embedding [61,62]. Researchers are still struggling to understand precisely how these mechanisms operate, or why they achieve such high performances. Might these ideas actually depart from computational principles common across neural systems? While this possibility remains open, note that a hierarchical mode of operation seems a constant even in the most novel architectures.

What fundamental mathematical principles might underlie the evolutionary convergences just outlined? A possibility is that both brains and CNN are tapping into some essential, objective structure of input stimuli. Using models of interacting spins, Stephens et al. [63] derived effective *statistical physics* of natural images, and found that edge-detecting filters (such as the ones in the retina and early layers of CNN) are the simplest, most salient features. Might similar statistical relevance in input signals explain successive layers as we trade simplicity for salience? A way to test this is by applying Stephens’s approach repeatedly, at several levels of the visual hierarchy—as implemented, e.g., for language features [64].

### 2.3. Wiring Cost Universals

A very different kind of convergent design involves the presence of optimal wiring principles in both brain and very large scale integrated (VLSI) circuits. Vertebrate brains, and the human brain in particular, are equipped with a very efficient architecture under strong packing constraints [65] that are followed by brains [66]. This close relationship between integrated circuits and neural systems is provided by the so-called Rent’s rule, which defines a power law in networks that exhibit hierarchical modularity. Assuming that we partition the system into sub-systems of size *N*, the rule establishes that the number of connections *C* linking the subset with the rest of the system scales as

(2) $$C=\langle k\rangle N^{p},$$

where 〈k〉 gives the average number of links per node, whereas 0≤p≤1 is the so-called *Rent’s exponent*. This is characteristic of fractal objects (where the basic pattern is repeated at different scales) and, thus, an indication of the presence of hierarchical order. When this method was applied to neural networks, a striking convergence was found. Both the neural web of the nematode *C. elegans* and human cortical maps shared a Rent’s exponent *p* close to what is the expected value for an optimally efficient hierarchical system. Such convergence that shares many properties in common with VLSI circuits, illustrates the role played by cost constraints in promoting convergent designs [67,68].

State-of-the-art design of hardware networks and microchips are pushing the limits regarding space and other constraints—e.g., some circuits must operate within tolerable latencies [69]. Machine learning techniques (e.g., reinforcement learning) might soon take over the manufacturing of new architectures [70]. Will these new generations of designs follow Rent’s rule as well? If this and other similar regularities emerge out of the underlying task (and not from the design process), we propose that convergences and (more interestingly) deviations from such laws would indicate whether the landscape of computational designs is being expanded in actual novel ways.

### 2.4. A Few Building Blocks of Dynamical Systems Enable Complex Cognition

Studying Recurrent Neural Networks (RNN) and certain cortical circuits as dynamical systems suggests that simple mathematical principles underlie complex cognition as well. Attractors, saddle nodes, and limit cycles of such complex, high-dimensional systems constitute a dynamic backbone that guides neural activity towards low-dimensional manifolds. Forces behind cognitive processes become readily accessible—thus opening “the black box” [71].

**Figure 3:**
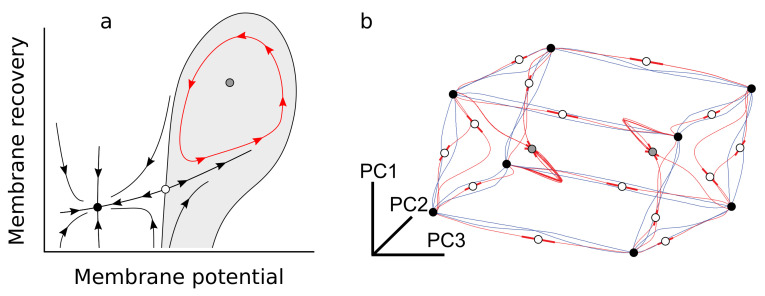
Attractors, saddle nodes, and repellers in the phase diagram of a dynamical system. (**a**) Phase diagram of a spiking neuron. A saddle node attracts trajectories along the vertical direction and splits the horizontal one in two diverging trajectories. Thus, it mediates the decision of whether to spike or not. (Plot inspired by [72]). (**b**) Attractors of the dynamics of a RNN partition the phase space into attractor basins that store $2^{3}$ bits of information. Saddle nodes mediate trajectories that diverge towards each attractor depending on the patterns of bits that need to be stored. (Panel adapted from [71]).

The phase diagram in Figure 3a shows an attractor (filled circle), a saddle node (empty circle), and an unstable fixed point (shaded circle) surrounded by a limit cycle (red). This depicts, qualitatively, the phase diagram of a leaky integrate-and-fire neuron. In real neurons, membrane potential is changed by currents injected from presynaptic axons, while recovery is driven by ion fluxes across the membrane that reset the neuron to its resting state (attractor). Noise or injected currents (of either sign) move the system around the attractor, sometimes towards the saddle node, which separates two dynamical regimes: at its left, the system returns to resting; at its right, dynamics are thrust around the limit cycle—a spike. Note how the dynamics around the saddle node are *attractive* along the vertical direction and repulsive along the horizontal one. For such integrator neurons, the saddle node mediates the “decision” of whether to spike or not by channeling trajectories along a single line that condenses all relevant information.

Early experiments on stimulation of real axons found that spiking behaviors always fell within a few classes [73,74]. It turns out that there are just a few ways in which attractors, saddle nodes, and limit cycles can interact in the phase diagrams of spiking neurons [72,75,76,77,78]. These explain integrator and resonator neurons (the later responding latency, rather than intensity, of input pulses), which pretty much exhaust the range of observed spiking classes [72]. Such simple decisions (whether to spike, whether to do it repeatedly, whether to do it at a fixed or variable firing rate) are thus mediated by a small dynamical alphabet.

Sussillo and Barak (2013) studied whether similar elements mediate decision making in RNN trained to perform different tasks. These networks are much more complex that spiking neurons, and their activity moves around high-dimensional spaces. They localized fixed points (both attractors and saddle nodes) numerically, and found that they partition the phase space according to the learned task. Figure 3b shows the backbone of an RNN that stores the last input (+1 or −1) of three independent neurons (thus, it must store $2^{3}$ patterns, each in a different attractor). In these and other RNNs, saddle nodes tend to be stable along most orthogonal directions except one. Thus, as for the integrator neuron, they channel all unimportant degrees of freedom towards one-dimensional trajectories (depicted in Figure 3b) that capture the relevant variables for the cognitive process.

Similar principles underlie some decision making processes in humans. Collective dynamics of cortical networks appear confined to low-dimensional manifolds [9,79,80,81], suggesting a channeling of activity towards useful cognitive regions. By combining neural data on a context-dependent decision task with RNN models, similar dynamical backbones can be uncovered [82,83].

### 2.5. Learning, Learning to Learn—Meta-Learning

Learning is a problem in which we saw impressive, yet irregular progress. The puzzle shows two distinct scales, from the microscopic to overarching principles, which we cannot fit yet in an all-encompassing picture. We know the details of memory formation in individual synapses [84], which build associations using Hebbian (”fire together, wire together”) reinforcement [85]. We also know several algorithms to implement similar plasticity in formal models of neural networks, with different strategies often giving rise to distinct cognitive frameworks [12,13,86]. In these algorithms, global information about system (brain or ANN) performance is precisely deployed to individual synapses [87]. In the cornerstone *backpropagation algorithm* [86], the chain rule of the derivative computes the causal contribution of each synaptic weight towards a global output error—thus, connections can be precisely corrected. This method is incredibly successful, as shown by recent ANNs with super-human performance in complex games [88,89,90,91].

While we understand functioning microscopic mechanisms and overarching principles for learning, we do not comprehend how learning descends from the complex, symbolic framework to the synaptic scale. There are serious issues concerning how the brain could implement the algorithms that solve these problems in machines [87]. Naive backpropagation would demand exact, symmetric backward paths to every synapse. Recent advances suggest solutions enabled by the generalizing and plastic abilities of the brain [87,92]. Under certain circumstances, if we deliver *fake error derivatives* (as a fixed, randomly weighted combination of global errors) to synapses, they can first adapt themselves to making these randomly weighted signals useful, and then proceed with learning as usual [87,93].

Problems become more pressing in Reinforcement Learning (RL) [94]. In RL, scenarios and rewards change as a response to agent actions. Feedback might arrive late in time. The problem of credit assignment affects not only individual synapses, but also representations of earlier, fading states. To implement RL, some form of time travel (see below), even if deeply unconscious, must take place. Evidence shows that, in mice, hippocampal place cells replay recent walks through a maze in reverse—a purported mechanism (and elegant solution) to assign credit to all states that contributed to the eventual reward [95,96,97].

The prefrontal cortex (PFC) seems a paramount site of RL in humans brains. Within a broader network (including basal ganglia and the thalamus), PFC implements meta-learning and generalization by combining two coupled loops of RL [98,99]. An outer RL loop allows PFC to learn broad classes of models, while the internal loop allows PFC to converge upon a model within the class that explains current input. This convergence happens within very few examples—also for inputs derived from models within the family but not previously shown to the network. Furthermore, this convergence happens without further modifications to PFC weights. This implies that memory about a current model is not stored as synapse changes, but as fading states of the network dynamics. Because of this, this mode of learning happens at the speed of network operation, not at the slower rate of network learning.

## 3. The Gap

The goal of building an artificial intelligence, as defined by the founders of the field, is far from being achieved. Great expectations were generated by breakthroughs in a diverse range of domains involving specific problem-solving. However, the achievement of human-like intelligence, or some alternative cognitive system able to deal with some of the crucial ingredients associated to the gap, is far from close. Larger multilayered networks are not enough to get there, and one direction to look has to do with our initial discussion concerning evolutionary convergence. In this context, although evolutionary algorithms are used in A.I., evolutionary arguments are too often ignored within the A.I. community. In this section, we consider some key qualitative traits that make human minds singular [100], and show how these features might constrain machine intelligence expectations. Table 1 summarizes the most salient similarities and differences, thus outlining our comparative approach to brains versus machine evolution.

### 3.1. Language

Language is a complex system itself, and has a network organization that includes multiple interacting layers [101,102]. Other species posses complex communication systems, but none of those shows recursivity—actually, no other species seems able to process recursively organized sequences [103]. This feature ultimately confers open-ended expressive capabilities to human language [104].

It has been argued that language has not evolved as a communication means, but as an advanced representation system [105,106]. This might have been triggered when facing, with an advanced brain, some evolutionary pressures common to other eusocial animals [107]. Such pressures would demand displacement: the ability of a signal to represent arbitrary events not immediately present. This problem is solved by many species (e.g., ant pheromones indicate a non-present reward), but Bickerton arguedthat this feature, planted in the much richer hominid brain, would kick-start an irreversible process towards full-fledged language. Alternative, more gradualistic views of language evolution assign perhaps even greater roles to evolutionary pressures [105,108,109,110].

The final stage of language is irreducibly complex, and it hardly leaves intermediate fossils in evolution nor in development. The closest to a language fossil is a debated *proto-language* form that arises in individuals who are trained late, or that emerges as a chimera of co-habitating tongues [104,107]. However, this seems an unstable cognitive solution: children of proto-language speakers readily complete it into a full-fledged language. This again suggests an irreversible evolution as language complexity crossed some threshold—as suggested by Bickerton.

As it evolved, language co-opted, or tapped into 15 circuitry for sequence representation [103]; auditory processing, including complex stimuli such as music [111] and motor control [109,110,112], among others [105,107]. It also sprawled a semantic network present all across the neocortex [113,114]. The most prominent regions for language implementation sit usually at the left hemisphere, around the Sylvian fissure [115,116,117,118,119,120], thus have ready access to vital cortices (auditory, motor, etc.). This neural substrate appears to be very similar across individuals and languages [121]. Besides these commonalities in their neural implementation, different tongues seem to share other universal traits such as their productivity [122] and some accounts of efficiency [123]. Notwithstanding the purported universalities, linguistic codes throughout the world present an astonishing variety [124].

Another feature common to all tongues across this huge diversity is ambiguity—a rather counter-intuitive trait. Animal communication codes are not ambiguous, as mistaken calls can be fatal [104]. Computer languages cannot accept polysemous instructions either. And yet, ambiguity is ever present in human language [125]. A minimal model of communication codes that simultaneously optimizes conflicting features suggests that ambiguity enables large expressive power with smaller vocabularies [126,127].

Ambiguity also enables semantic accessibility. Semantic networks connect words that are related through their meaning (usually, by being synonyms). They can be derived, e.g., from curated linguistic corpora [127,128,129] or from free-association experiments [130]. Semantic networks present a scale-free and small-world structure *provided that polysemy is included*. Scale-free graphs are dominated by a few large hubs (central concepts that link to many others), while most words only have a few connections. This places some constraints on how language webs can be implemented in neural hardware [102,131], suggesting that a statistical regularity hides a relevant constraint of language evolution. Small world is defined by networks with a high clustering (i.e., abundant triplets of interrelated concepts—thus having abundant local connections) and small average distance between nodes. Thus, polysemy makes semantic networks easy to navigate through a search by association [125,132].

Most approaches to implement artificial language attempt to manually hard-wire some overall computational, syntactic traits, or to infer grammars from large corpora. However, alternatives exist that take seriously the relevance of Darwinian evolution in the origins of language. Notably, Luc Steels’s *Talking Heads* experiment [133,134,135,136]) allowed to develop setups with embodied robots that converse about an external world. Steels capitalized on Fluid Construction Grammars [137], a framework that includes ambiguity while managing combinatorial explosions—a key aspect of syntax. As robots exchange appreciations about their external world, their grammars, syntax, and semantics mature and their understanding of the environment becomes sharper.

It might be possible to build human-like language by design. However, if Bickerton’s suggestion of an irreversible evolution under the appropriate circumstances are true, setting up evolutionary frameworks for artificial minds might ease the work. Alternatively, since artificial cognitive systems are different from humans, we can wonder what effects such evolutionary pressures might have on them—what kinds of communicative or representation systems such dynamics might bring about.

### 3.2. Time Travel

We are *time travellers*, able to locate ourselves in time by storing past events in multiple layers of detail while being able to imagine multiple alternative futures [138]. The past is reached thanks to episodic memory (which is autobiographical in nature) and mixed evidence suggests that animals might have a rather limited capacity to remember personal episodes [139,140]. No current artificial system has this ability; although many features such as goal-directed behavior, planning or causation require time representation.

The powerful capacity of brains to explore possible futures is not reducible to simple rules of forecasting, which were likely present in early stages of brain evolution. This ability seems enhanced (if not enabled) by the language capacity—the displacement property (to represent scenarios not currently available) is naturally extended into imagining futures. While non-human animals have a limited ability to plan the future, it does not come close to the human capacity that language brings about. In evolutionary terms, predicting future events has been a major force towards cognitive complexity: reducing environmental uncertainty can largely counterbalance the costs of a cognitive apparatus [5,6]. Past recollection and generation of possible futures seem intimately connected, as the same areas that are used to recall past events have been co-opted to plan future events and ultimately to create alternative futures [141].

So far, the time dimension of cognition is barely represented within neurorobotics, where research focuses mainly on the spatial extent of sensory information. The reason is the preeminent role played by information processing associated to sensory devices, whereas the role of time is limited to find out the next possible action. Implementing temporal cognition is being recognized as a missing component in neurorobotics [142]. In any case, early work on time representation in connectionist models already indicated that recurrent networks might be a necessary condition [143] and a precursor component of complex language is used by a symbolic mind.

We expect Reinforcement Learning to be the branch that most early explores time traveling—as policies extend in time. In recent breakthroughs, RL agents first elaborate internal representations of their external world [144,145]. This allows a limited forecasting, and even *dreaming* worlds over longer periods [144]. This way, policies are better informed, improving performance. These models based their internal representations (and limited time travel) in the simplest correlations between sensory information (pixels in a screen over time). Meanwhile, human mental models include proper objects and agents causally related. It is the extrapolation of such causal relationships that enable our rich time travel experience.

### 3.3. Mind Reading

We can identify emotions and states of mind of others thanks to a set of specialized systems that evolved as part of our lineage’s social nature. Face recognition processes are devoted ample and specialized regions in our brain [146], along with a system of mirror neurons that respond to actions of others as if they were our own [109,147,148]. Although mirror neurons are shared with other species, the consequences for humans are enormous. The capacity for reading minds was a crucial component in our evolution as a cooperative species: knowing the mind of others provides an effective way of making decisions relevant to group needs. In addition, such mechanisms, likely to be the target of evolution, open the door to another remarkable trait: self-consciousness. Developing a theory of mind might inevitably create the conditions for “knowing” your own individual, distinct nature. In this view, self-consciousness would be a side effect of mind reading.

Thus, here too, evolutionary dynamics has played a central role in developing mechanisms for social interactions that are likely to be a major requirement to develop advanced artificial cognition. So far, emotions are detected by robotic agents able of visual pattern recognition and supervised learning. If evolution and social interactions are a requirement for self-awareness, the often discussed possibility of generating conscious A.I. might necessarily demand interactions among communicating agents. Within robotics, this requires the generation of internal representations that encode information about internal states of other agents—i.e., about information of the external world that is not readily available to sensory systems.

### 3.4. Right from Wrong

We have a moral mind, and evidence indicates that there is some hardwired tendency to make the right moral decisions early on in life. Cooperating moral minds have been helpful in fostering communities, and thus generate meaning under the context of social interactions [149].

Building moral machines is a hot topic within both A.I. and robotics. Here, too, moral brains are evolved systems [150,151], whereas machines require an explicit programming of moral norms [152]. While the goal of evolving machines that will avoid harming humans has been a recurrent topic within fictional works such as Asimov’s rules of robotics (As noted by Brooks (2003) and Mitchell (2019), Asimov’s Three Rules of Robotics illustrate the nontrivial character of moral decisions. Because of the importance of context (or the environment) apparently well-established programmed rules can conflict with each other and create unexpected, and sometimes undesirable outcomes), it becomes a pressing issue as autonomous robots are being deployed [153]. This connects inevitably with time travel and mind reading: moral decisions imply choices and understanding their implications for others. That means having a theory of mind, representing counterfactuals, future paths (as it occurs with the famous trolley experiment), and the implications of these actions.

### 3.5. Extended Mind

A major ingredient for the success of humans in their ecological conquest of the biosphere is related to their remarkable capacity for understanding and manipulating their environments. We are somehow limited by our senses or physiology, but none of these limitations really matters, since all of them can be overcome by the appropriate technological interface. This is part of what Clark and Chalmers dubbed *the extended mind* [154]. Extended cognition starts with the embodied nature of agents and ends in the specific interfaces that they use to connect with their worlds. It can be found in very simple organisms. One very interesting example is provided by spiders, as their spiderwebs define a powerful example of niche-constructed structures that outsource information processing by means of an externalized structure [155]. In this case where small brains are involved, external structures allow to reduce environmental uncertainty. Insect nests would be another successful example [156]. In this case, nests act as both engineered structures regulating from self-organization and a vehicle for ecological engineering.

Little is found in their synthetic counterparts: beyond embodiment, robot-driven manipulation of their environments is almost absent. In silico counterparts based on reinforcement learning are the closest experiments in this direction, although limited to simulated environments [157,158]. A very recent, promising proposal uses the web as an embodied environment to improve artificial question-answering [159].

### 3.6. Social Learning

Thanks to language and mind reading, and fostered by extended cognition, humans massively involve themselves in complex social interactions. One particularly relevant aspect of this is social learning: the extraordinary capacity to learn from others and being able to transmit information through teaching. A great deal of this is connected with imitation, which is unevenly distributed in non-human vertebrates. Songbirds or cetaceans display high levels of imitation, while non-human primates have rather limited skills [106]. Some authors suggested that this is a crucial attribute that is needed to create true human-like machines [160]. Social learning has been a very active domain within robotics, particularly within the context of human–robot interactions. A broad range of social robot scenarios can be defined [161], from ant-like robots to potential socially-intelligent agents (the latter within the domain of speculation). A specially relevant development in this area deals with the design of human-shaped robots able to learn facial expressions and react to them in meaningful ways [162,163].

What is required to move forward beyond imitation and emotion detection? Here, we might need mind-reading features and embodied interactions in order to properly create complex social exchanges. An important contribution in this direction is the work of Arbib and co-workers aimed at building agents that are explicitly equipped with a mirror neuron system [164]. In their model, which avoids the extra complexities of language, they considered gestures as the communication channel between virtual simulations of interacting agents. In this *dyadic* brain model, two agents interact by combining both (simulated) visual and manual interactions. In a nutshell, the model allows two agents to learn how to exchange, predict and react to each other’s gestures in a ritualized manner. These are very promising steps towards linking social learning with mind reading. As suggested by some authors, these might have been crucial ingredients for major cognitive transitions in human evolution [163,165], and the transition would be, once again, a qualitative shift.

We expect progress in ‘mind reading’, ‘right from wrong’, and ‘social learning’ to go hand by hand, as some of these lines can be preconditions or even foster advances for others. While there is still a relevant gap to the complexity of these traits in humans, some progress is being made with agents that use reinforcement learning in simulated games or virtual environments [166,167,168].

## 4. A Space of Cognitive Complexity

The set of properties displayed by human brains that define a cognitive gap can be represented in a unified way by means of a *morphospace*—i.e., a three-dimensional space that allows a mapping of all given case studies within a finite domain. By using such a space, we can locate the different systems and compare them. Additionally, the presence of voids (i.e., empty volumes lacking any candidate system) can provide evidence for constraints or forbidden evolutionary paths. This approach was first introduced within the context of morphological traits of shells [169] and has been later on widely used within Paleobiology and evolutionary biology [21,170,171,172], and in other different contexts including network science [173,174,175] and computational neuroscience [176,177,178,179,180,181]. Morphospaces provide us with a global picture of possible designs and how they relate to each other (whether they are distant or close) in a feature space. By making reasonable assumptions about relationships between features, we can still make some qualitative assessments about our systems of interest [176,179,180,182].

**Figure 4:**
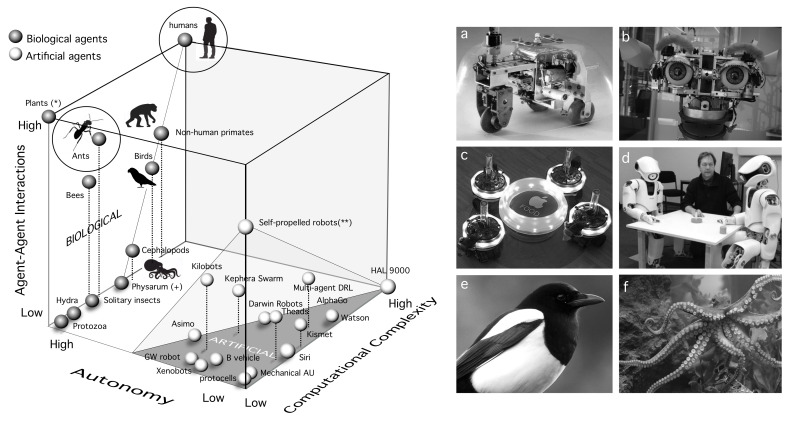
A Morphospace of cognitive complexity. Autonomous, computational, and social complexities constitute the three axes of this space. Human brains are located in the upper corner, scoring with maximal autonomy, computational complexity, and agency. The examples shown here include both natural and artificial systems, as indicated. Plants (*) are located in the upper left corner since ecological interactions are known to play a key role, some of them by means of chemical communication exchanges. Current A.I. implementations cluster together in the high-computation and low-social complexity regime, with variable degrees of interaction-based rules (as it occurs with multiagent Deep Reinforcement learning, DRL). Simple embodied systems displaying low computational complexity include mechanical automata, xenobots or Braitenberg vehicles. Another limit case here is provided by self-propelled robots (**) which are randomly moving, bullet-shaped agents carrying no sensors nor internal states, that interact physically leading sometimes to collective swarming patterns. The boundaries of this artificial subset (dark gray) are limited in the Autonomy direction by a boundary where several instances of mobile neurorobotic agents are located (such as Asimo, Kephera robots or different versions of robots build by Gerald Edelman and collaborators). The left wall of high autonomy is occupied by living systems displaying diverse levels of social complexity. This includes some unique species such as Physarum (+) that involves a single-celled individual. On the right, six different particular case studies are highlighted, namely: (**a**) Gray–Walter tortoise (a simple cybernetic mobile robot), (**b**) Kismet social robot able to detect and respond to emotions from visual cues, (**c**) swarms of Kephera robots with evolvable neural networks, sensors, and lights to communicate information, (**d**) talking heads experiment using humanoid robots (image courtesy of Luc Steels, in the image). Two examples of animal minds that share common complexities with human behavior, despite their marked differences in brain architecture are (**e**) magpies and (**f**) octopuses (image by Kelly Tarlton).

A morphospace of cognitive complexity is outlined in Figure 4. We propose three axes:

1. **Computational complexity**: This needs to be understood as some measure over the tasks performed by each kind of agent. That would include memory, learning, decision making, and other cognitive traits.
2. **Degree of autonomy**: This is a crucial attribute of adaptive complexity. We can define autonomy as “the property of a system that builds and actively maintains the rules that define itself, as well as the way it behaves in the world” [183].
3. **Interactions between agents**: This third and no less relevant dimension might enable cognition capabilities that transcend the individual. Tight interactions between agents might be a pre-requisite for (or a consequence of) eusociality [184], as they might enable a switch of the selective focus of Darwinian selection.

At the bottom-front of the morphospace, we locate the region where artificial systems exist, whereas most living organisms populate the left vertical, high-autonomy wall. The bottom surface of this space includes all those systems that lack the social component—they need little interaction with others to sprawl their cognitive phenotype. Here, we have several kinds of robots as well as mechanical automata, protocells, and solitary organisms. A most obvious feature of our plot is that living and artificial systems appear separated by a gap that grows bigger as systems become more complex or more socially interactive. The divide reflects a fundamental difference between biological and artificial systems: the pressure of Darwinian selection and evolution that promotes autonomy (as discussed in [185] in terms of selfishness) [182,186]. Composed replicative units are more complex, thus can support the propagation of their selves with enhanced internal computation that enables to predict ever more complex environments [5]. Due to evolution, this computational prowess must further protect autonomy—thus closing a reinforcing loop that necessarily pushes biological replicators towards the left wall of our morphospace.

Most artificial agents have not come together through Darwinian selection. Instead, they are typically designed or programmed to perform functions under environment-free scenarios, with some exceptions. The closest to large autonomy are embodied neurorobots (again, embodiment as a driver for true cognitive complexity) that are capable of sensing external cues, move in space and react in simple ways. Gray–Walter’s tortoise or Braitenberg’s vehicles (GW robot and B vehicle in Figure 4, left) are early precursors: they are electromechanical robots with a minimal sensorimotor architecture that allows them to respond to simple cues (approaching or avoiding lights or returning to the charging source). One great leap has been provided by the development of robots able to learn through experience using ANN, combining movement, visual processing, and motor responses (Darwin robots in particular have been developed under a set of design principles that are inspired in cortical architectures. In their implementation, these simulated cortical areas mimic reentrant neuroanatomic connections (an important feature that pervades high-level brain properties, including consciousness). Moreover, each area contains neuronal units that represent both activity levels and the timing of the activity of groups of neurons. As it occurs with real brains, neuronal synchronization allows neural binding) [187].

As we move up from this surface and consider the role played by agent interactions, we also see a rather uneven distribution of case studies. Within the artificial domain, with few exceptions, interactions are limited to some particular features associated to the way they have been trained to perform very specific types of tasks (such as playing games). Two important exceptions are Luc Steel’s Talking Heads [135] and swarm robotic systems such as Kilobots [188,189]. In the latter, computational complexity is externalized while autonomy is required to making decisions associated to their relative location to others. One corner in this domain involves self-propelled robots that have been extensively studied as a class of active matter [190]. They have zero computational complexity (they move by means of vibrations) and their random behavior in isolation discards non-zero autonomy since they have no real sensing of the external world. Another, isolated corner is represented by fictional entities (such as Asimov’s robots or HAL9000) that would be pre-programmed to behave as intelligent agents without being exposed to social interactions.

By contrast, life covers the left wall by a diverse range of possible combinations of computational complexity and social computation. The bottom of the wall, as we pointed out before, is occupied by individual-based life styles, including single-cell species (protozoa or Physarum), Hydra or solitary insects. As we move up in the social axis, two major winners in the competition are social insects and humans. They have both successfully conquered the planet [184] thanks to their enormous adaptive potential, although the cognitive complexity of individuals is markedly different [191,192]. Collective intelligence in social insects results from parallel, spatially-extended interactions between individuals that can be blind and exhibit a very poor individual repertoire in isolation. What about other animals? Mounting evidence indicates that there is a continuum of both social and cognitive complexity that allows to tentatively allocate different groups (again, this is a coarse-grained picture, with considerable variability) equipped with diverse neural apparatuses [193] and displaying a varying degree of social complexity. Once again, humans depart from them in a rather singular way: they are individually complex and able to massively engineer their environments by means of the extended mind resulting from cultural evolution, technology, and information.

**Figure 5:**
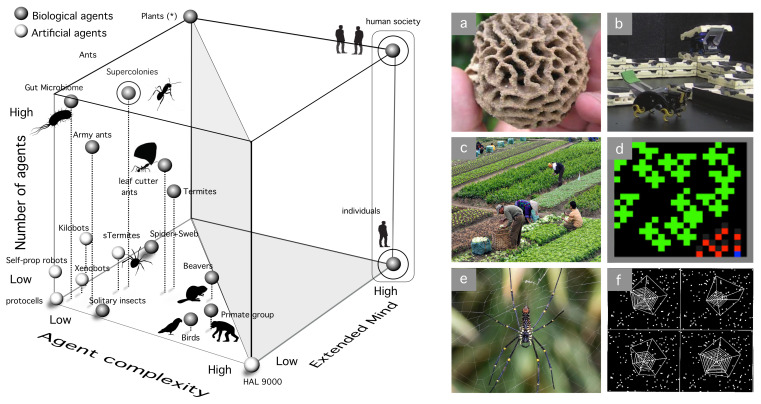
A Morphospace of extended minds. A space of cognitive agents (**left**) can also be constructed by considering again the agent complexity axis (as in the previous figure) along with the number of agents involved in a given group as well as the role played by extended cognition (EC). The latter includes the presence of active mechanisms of niche construction, ecosystem engineering or technological skills. The corner occupied by plants (*) involves (for example, in a forest) small computational power, massive populations and an “extended mind” that needs to be understood in terms of their interconnectedness and active modification of their environments, particularly soils. While ants in particular rival humans in their shear numbers and ecological dominance, the massive role played by externalized technology in humans makes them occupy a distinct, isolated domain in our cube (with both individuals and groups able to exploit EC). Some remarkable examples of EC are displayed on the right panels. Termites create complex spatial structures (**a**) fungus-growing chamber) and have inspired some swarm robotic systems (**b**) able to construct structures. Farming has been one of the ways humans have engineered their environments (**c**), and some deep multiagent reinforcement learning models (**d**) show how a collective of agents interacting with a given, limited resource can give rise to cooperative strategies. The dominant role of EC in some species beyond humans is illustrated by cobweb spiders (**e**). They are equipped with a tiny brain, but their spiderwebs act as sophisticated antennas, which allow for a powerful sensing and response to their environments. In this context, efficient cobwebs can be evolved using artificial evolution (**f**). The gap separating humans from the rest of natural and artificial systems is highlighted by the empty volume on the right, which needs to be explored by future models of artificial cognition.

This departure is highlighted in Figure 5, where an additional space of possible cognitions is shown. Here the cognitive complexity dimension is completed by system size (how many agents define the group) and the role played by extended cognition (EC). Here, we move beyond the boundaries of single multicellular agents and consider groups of agents of diverse kinds, from small primate groups to the massive, multi-queen ant colonies known as “supercolonies”. The case study of ants is particularly relevant in terms of their ecological impact on the biosphere by means of an active modification of their environments, only comparable to the impact of humans. As E.O. Wilson pointed out, had humans failed to colonize the planet, the biosphere would be dominated by social insects [184]. However, in stark contrast with human or vertebrate brains (see Table 1), ants are equipped with small brains and the cognitive power comes from the *collective intelligence* resulting from agent–agent interactions as well as with their capacity to build large-scale structures (Figure 5a) that are several orders of magnitude larger than the individual size. These have inspired the development of simple robotic agents that build structures, such as the artificial termites (eTermites in the morphospace) in Figure 5b [194]. Humans, on the other hand, can act as ecosystem engineers and exploit their EC on multiple scales, from single individuals to large collectives. The large, empty void in the space is a reminder of the enormous distance taken by humans in relation to any other species, as well as the lack of machine learning models of agents displaying EC. Some steps in this direction have been made, which are inspired in some key examples such as framing (Figure 5c). Using a deep reinforcement learning system, a set of agents can discover rules of cooperation that might recapitulate the early steps towards managing ecosystems before the emergence of agriculture [195] (Figure 5d). Finally, one remarkable example of EC is provided by the spiderwebs created by spiders having very small brains (Figure 5e) that act as effective auditory sensors with a total surface that is $10^{4}$ times larger than the individual spider. By building this externalized cognitive apparatus, individuals are released from body size constraints [196]. Can these kind of structures (rather special mind extensions within biology) be generated by an evolutionary model of spider behavior (Figure 5f) but require to define a priori some constraints related to the types of rules required to generate the web [197]? Here again, the evolutionary emergence of the innovation represented by the spiderweb is far from the current state of the art of A.I. Crossing the empty land with two cognitive agents displaying complex extended mind remains a challenge.

Solving the problem of constructing a true A.I., as suggested by the structure of the morphospace, will require much more than cumulative innovations. As it occurs with evolution, new innovations might require major evolutionary transitions [18]. These results are further expanded and summarized in Table 1, where different features associated to cognitive complexity are presented for the main four groups of systems discussed here, namely human and non-human vertebrate brains as well as deep networks and neurorobotic agents.

## 5. Discussion

Can machines ever achieve true intelligence? In a recent paper entitled “Building machines that learn and think like people” [198], it has been argued that, for ANN to rapidly acquire generalization capacities through learning-to-learn, some important components are missing. One is to generate context and improve learning by building internal models of intuitive physics. Secondly, intuitive psychology is also proposed as a natural feature present since early childhood (children naturally distinguish living from inanimate objects) which could be obtained by introducing a number of Bayesian approximations. Finally, compositionality is added as a way to avoid combinatorial explosions. In their review, Lake et al. discussed these improvements within the context of deep networks and problem-solving for video games, and thus considered the programming of primitives that enrich the internal degrees of freedom of the ANN. These components would expand the flexibility of deep nets towards comprehending causality. (See also the life-long work of Jürgen Schmidhuber for important developments over the last decades in the meta-learning or learning-to-learn paradigms: https://people.idsia.ch/~juergen/metalearning.html, accessed on 2 May 2022). Lake et al. also pointed at several crucial elements that need to be incorporated, being language a prominent one. So far, despite groundbreaking advances in language processing, the computational counterparts of human language are very far from true language abilities. These improvements will without doubt create better imitations of thinking, but they are outside an embodied world where—we believe—true complex minds can emerge by evolution.

Are there alien minds? Yes and no. An affirmative answer emerges from the obvious: artificial systems do not need to follow biological constraints or Darwinian evolutionary paths. Being designed by humans or evolved within computers using ad hoc optimization procedures, the final outcome can depart from biology in multiple ways. A deep network can outperform humans in a very specific task using a training algorithm based on feed-forward convolutional nets that, although inspired by experiments, lack the re-entrant loops that might be crucial to achieve true intelligence and awareness. Robotic agents can have behavioral patterns of response to complex environments, but the cognitive skills are externalized: algorithms are being executed in a rather powerful computer that resides somewhere else outside the body. However, these are systems where agency plays a minor role. Perhaps, the really relevant question is this: are there autonomous alien minds?

If convergent designs are an indication that there is a limited repertoire of possible neural architectures and cognitive autonomous agents, the future of A.I. is in the evolutionary arena. That means that the roads not taken necessarily cross the land of embodiment: as it occurs with naturally evolved systems, moving in an uncertain world was one of the engines of brain evolution. Moreover, another crucial component of evolutionary innovations is the emergence of new forms of cooperation. Cognitive agents mean evolving communication and dealing with information [193]. What kind of interesting phenomena can be observed using these two ingredients? Evolved robotic systems illustrate fairly well the ways in which evolutionary dynamics simultaneously link some of these components of cognition. As an example, robotic agents moving on a landscape where both positive and negative inputs (sources of charge and discharge, respectively) are located on given spots develop communication along with cooperative strategies that improve group fitness [199,200]. Each robot is equipped with a set of sensors and lights and start foraging with a random configuration. A feedforward ANN allows evolving the interactions between sensors and lights and to generate communication among robots that allows for cooperation and altruism. Finding and avoiding positive and negative scenarios create the conditions for increasing group fitness. However, crowding also triggers cheating and deception (a familiar trait of evolution): robots can also evolve into lying to each other. Despite the simple nature of the players, a combination of some key evolvable features can lead to unexpected insights.

As pointed out in the Introduction, the paths that lead to brains seem to exploit common, perhaps universal properties of a handful of design principles and are deeply limited by architectural and dynamical constraints. Is it possible to create artificial minds using completely different design principles, without threshold units, multilayer architectures or sensory systems such as those that we know? Since millions of years of evolution have led, through independent trajectories, to diverse brain architectures and yet not really different minds, we need to ask if the convergent designs are just accidents or perhaps the result of our constrained potential for engineering designs. Within the context of developmental constraints, the evolutionary biologist Pere Alberch wrote a landmark essay that can further illustrate our point [201]. It was entitled “The Logic of Monsters” and presented compelling evidence that, even within the domain of teratologies, it is possible to perceive an underlying organization: far from a completely arbitrary universe of possibilities (Since failed embryos are not the subject of selection pressures, it can be argued that all kinds of arbitrary morphological “solutions” could be observed), there is a deep order that allows to define a taxonomy of “anomalies”. Within our context, it would mean that the universe of alien minds might be also deeply limited.

## Figures and Tables

**Table 1:** Comparative analysis of human and non-human (NH) vertebrate brains, standard deep artificial neural networks, and evolved neurorobotic agents. This table highlights the current chasm separating living brains from their computational counterparts. Each item in the non-human is intended to reflect a characteristic quality, which does not reflect the whole variability of this group (which is very broad). For the DANN and robotics columns, there is also large variability and our choice highlights the presence of the given property at least in one instance. As an example, the wiring of neural networks in neurobotic agents is very often feedforward, but the most interesting cases studies discussed here incorporate cortical-like, reentrant networks.

	Human Brains	NH Vertebrate Brains	Deep AN Networks	EVOL-Neurorobotics
Wiring	Hierarchical-nested	Hierarchical-nested	Feed-forward	FF, programmed
Basic units	Neurons	Neurons	Threshold units	Threshold units
Internal dynamics	Critical	Critical	Point attractors	Sensorimotor control
Time travel	Yes	Limited	None	None
Generalisation	Yes	Limited	No	No
Language	Syntactic	Simple	None	Proto-grammar
Meta-learning	Yes	Limited	Learning To learn	None
Mind readers	Yes	Limited	No	Emotion detector
Right ≠ wrong	Yes	Yes	Built Ethics	Built Ethics
Extended mind	Vast	Limited	No	Embodiment
Social Learning	Dominant	Limited	No	Imitation learning

## Data Availability

Not applicable.
